# Phylogroup Homeostasis of *Escherichia coli* in the Human Gut Reflects the Physiological State of the Host

**DOI:** 10.3390/microorganisms13071584

**Published:** 2025-07-04

**Authors:** Maria S. Frolova, Sergey S. Kiselev, Valery V. Panyukov, Olga N. Ozoline

**Affiliations:** 1 Department of Functional Genomics of Prokaryotes, Institute of Cell Biophysics of the Russian Academy of Sciences, Federal Research Center “Pushchino Scientific Center for Biological Research of the Russian Academy of Sciences”, 142290 Pushchino, Russia; anthyllium@gmail.com; 2 Department of Bioinformatics, Institute of Mathematical Problems of Biology RAS—The Branch of Keldysh Institute of Applied Mathematics of Russian Academy of Science, 142290 Pushchino, Russia; panyukov@itaec.ru

**Keywords:** *E. coli* phylogroups, *k*-mer-based intraspecific phylotyping, interspecific relationships, bimodality, colorectal disorders, antibiotic treatment, probiotic therapy, obesity, Mediterranean diet, virtual diagnostic

## Abstract

The advent of alignment-free *k*-mer barcoding has revolutionized taxonomic analysis, enabling bacterial identification at phylogroup resolution within natural communities. We applied this approach to characterize *Escherichia coli* intraspecific diversity in human gut microbiomes using publicly available datasets representing diverse human physiological states. By estimating the relative abundance of eight *E. coli* phylogroups defined by their 18-mer markers in 558 fecal samples, we compared their distribution between gut microbiomes of healthy individuals, patients with chronic bowel diseases and volunteers subjected to various external interventions. Across all datasets, phylogroups exhibited bidirectional abundance shifts in response to host physiological changes, indicating an inherent bimodality in their adaptive strategies. Correlation analysis of phylogroup persistence revealed positive intraspecific connectivity networks and dependence of their patterns on both acute interventions like antibiotic or probiotic treatment and chronic bowel disorders. Along with predominantly negative correlations with *Bacteroides*, we observed a transition from positive to negative associations with *Prevotella* in *Prevotella*-rich microbiomes. Several interspecific correlations individually established by *E. coli* phylogroups with dominant taxa suggest their potential role in shaping intraspecific networks. Machine learning techniques statistically confirmed an ability of phylogroup patterns to discriminate the physiological state of the host and virtual diagnostic assays opened a way to optimize intraspecific phylotyping for medical applications.

## 1. Introduction

*Escherichia coli* (*E. coli*) is a facultative anaerobic bacterium commonly found in the intestinal microbiota [1] and is involved in maintaining gut microbiome homeostasis [2]. Advances in genomic sequencing and phylotyping have led to the progressive classification of *E. coli* into increasingly refined phylogenetic groups, initially categorized into four [3], then five [4], seven [5,6], eight [7,8,9,10] and, most recently, twelve phylogroups [11]. For intraspecific classification, two main approaches have been widely used: multilocus sequence typing (MLST) [5,7] and a PCR-based method developed by Clermont and colleagues [3,4,6,8], which targets virulence-associated genes. These studies have demonstrated that pathogenic *E. coli* strains are not randomly distributed across phylogroups [12], leading to the general assumption that pathogenicity emerging in some phylotypes due to horizontal transfer of virulent genes [13] requires a specific genetic background for their expression [14]. Consequently, phylotyping has gained significant importance in epidemiological surveillance and pathogenicity studies.

In 2018, in silico PCR-based classification and MASH clustering using marker *k*-mers [15] were combined in a new tool, ClermonTyping [6]. By analyzing a dataset of over 300 *E. coli* genomes, the authors observed only a few discrepancies between the two approaches: one relying on virulence gene detection and the other assessing genomic background. In our previous study [10], we compared MLST-based typing of 124 genomes with *k*-mer-based clustering and found that both methods generated topologically congruent trees, consistently identifying the eight established *E. coli* phylogroups (A, B1, B2, D, E, F, C and G). However, a subsequent MASH-based analysis of a much larger dataset, containing 10,667 *E. coli* and *Shigella* genomes, divided them into 12 clusters [11]. While phylogroups A, B1, F, C and G remained distinct, B2 was split into clusters B2-1 and B2-2; D diversified into subgroups D1, D2 and D3, while E separated into E1 and E2. Thus, despite the significantly expanded dataset, no entirely new phylogroups were discovered, suggesting that the eight major phylogroups represent the core genetic structure of *E. coli*.

While all phylogroups include strains prone to pathogenicity, epidemiological evi-dence indicates that ancient groups F and D harbor more pathogenic bacteria compared to groups A and B1 [9,16], whereas recently diverged lineages are frequently associated with severe infections [8,12,14]. A striking example is the 2011 German outbreak of *E. coli* infections, caused by the enteroaggregative hemorrhagic strain O104:H4 C227-11 [17,18,19], belonging to group B1. This strain exhibited exceptional virulence, attributed to the accu-mulation of horizontally acquired genetic determinants of pathogenicity [17,19,20,21]. These virulence factors included enhanced adhesion mediated by AAF plasmid-encoded fimbriae [19], potent cytotoxicity due to prophage-derived Shiga toxin (Stx-2) [17], and multidrug resistance mainly conferred by extended-spectrum β-lactamases.

Large phylogroup B1, which comprises about 30% of known *E. coli* genomes, includes only a small portion (2.4%) of potentially virulent strains, such as those of serotypes O104:H4 and O121:H19 [11]. In contrast, group E, currently comprising just 10.9% of known genomes, harbors a disproportionally high fraction (10.2%) of highly pathogenic *E. coli* strains of serotype O157:H7 [11]. The remarkable virulence of O157:H7 stems from extensive horizontal gene transfer, having incorporated genetic material from 53 species [22] and over 460 prophages [23] (compared to only 29 in non-pathogenic *E. coli* K-12 [24]). Major chromosomal pathogenicity factors include Shiga toxin-encoding prophages (similar to those in O104:H4) [25] and five LEE (locus of enterocyte effacement) pathogenicity islands, encoding a type III secretion system and adhesins [26]. Over 1000 genes are absent from non-pathogenic *E. coli* K-12 [27,28], forming about 180 O157:H7-specific O-islands [29], and including 131 potentially virulent genes [28]. Given these distinctive genomic features, phylogroup E strains are readily identifiable through both PCR-based typing and *k*-mer-based clustering [6,10,11].

The pathogenicity of B2 strains is strongly associated with the 54 kb *pks* genomic island, which encodes enzymes for colibactin biosynthesis [30,31]. However, the presence of such islands is not exclusive to pathogenic bacteria. Even the probiotic strain *E. coli* Nissle 1917, which is widely used to treat various intestinal disorders [32], has a *pks* island in the genome [33,34]. Nevertheless, neither live bacteria of this strain nor the spent culture supernatant had genotoxic effects [33], exemplifying epistatic suppression of virulence genes. While B2 is often considered as the most pathogenic phylogroup, its clinical prevalence may reflect enhanced ecological fitness rather than intrinsic virulence [35]. This is exemplified by CTX-M-15-producing B2 strains (serotypes O16:H5 and O25b:H4) that caused major outbreaks of infectious diseases in the early 2000s through their extended-spectrum β-lactamase-mediated antibiotic resistance [36,37]. Additional B2 virulence factors include cytolethal distending toxins (CDTs) causing DNA damage in infected cells [30] and cytotoxic necrotizing factor (CNF) [38]. Both types of genes are frequently plasmid-encoded but many are located within genomic islands [30,38,39], contributing to the genomic background traced by virtual *k*-mer-based screening of chromosomes.

The effectiveness of PCR-based phylotyping indicates that phylogroups have a specific combination of marker genes. This was recently validated in a comprehensive study of 844 uropathogenic *E. coli* strains, which revealed certain associations between phylogroups and specific virulence factors, including genes of antimicrobial resistance, motility and biofilm formation [40]. According to the efficiency of PCR typing, 111 strains of phylogroup G showed no isolates carrying genes for the adhesin Air, toxin Sat and the transcription factor EilA [9], of which Air and EilA are specific to groups D and F, while Sat is produced by group D and B2 bacteria [41]. Phylogroup A virulence is most often associated with the adhesive fimbriae FimA and YfcV, as well as receptors for yersiniabactin (FyuA) and ferric aerobactin (IutA) [42]. Isolates from the less studied group C encode the enzyme HlyF [43], which triggers eukaryotic autophagy due to toxin release via outer membrane vesicles [44]. In combination with the Shiga toxin of *E. coli* O80:H2, this caused bacteremia in Europe [45]. Therefore, not all but many virulence genes are distributed across most phylogroups (Shiga toxins Stx-1/2 so far have only not been found in group G genomes [46,47]). However, the mere presence of virulence genes does not guarantee pathogenicity. As proposed two decades ago, their functional integration requires a compatible genetic background [14].

Current understanding suggests that this genetic background emerges through complex interactions between cellular regulatory networks and horizontally acquired genes via epistatic relations. The enhanced recombination efficiency within phylogroups likely promotes the preferential maintenance of beneficial and/or virulence genes among phylogenetically related strains. Yet even phylogroups harboring multiple virulence determinants show unpredictable expansion patterns, implying the existence of more complex, higher-order epistatic interactions within microbial communities. To investigate this phenomenon at the level of *E. coli* phylogroup homeostasis, we employed *k*-mer-based profiling of natural microbiomes. The study specifically examined how endogenous and exogenous factors triggering adaptive responses in the human gut microbiome influence intraspecific equilibrium within *E. coli* populations.

## 2. Materials and Methods

### 2.1. Datasets Used for Intraspecific Taxonomic Analysis

The metagenomics data taken for this study were obtained from the European Nucleotide Archive (ENA), containing whole-genome sequencing (WGS) reads derived from human gut microbiomes (Table 1). The PRJEB7774 dataset, which includes samples from healthy volunteers and patients with colon adenoma and carcinoma [48], was chosen as representative of “stable” microbial communities that have adapted to distinct host physiological states. In contrast, the PRJEB28097 dataset comprised stool samples collected from healthy volunteers before and after the acute treatment with ciprofloxacin and metronidazole [49,50]. A previous study demonstrated that this combination of antimicrobials significantly reduced the abundance of all *L. paracasei* phylogroups [51], leading us to anticipate a similar response from other bacteria, including *E. coli*. The PRJEB33500 dataset contained fecal samples from overweight individuals, collected before and after adherence to a Mediterranean diet, which modulated gut microbiome composition [52]. This dataset was particularly valuable as it enabled direct comparison of intraspecific bacterial homeostasis caused by a relatively comfortable transition to healthier dietary patterns, representing a more physiologically gradual perturbation compared to antibiotic intervention or disease states.

Fastq files underwent pre-processing steps, such as removal of adapter sequences and quality control using Trim Galore v.0.6.10 [53]. Reads shorter than 20 nucleotides and low-quality reads with Phred scores below 20 were excluded from the analysis.

### 2.2. Genome Collections

Two genome sets were used in this study. Set 1 contained 124 genomes previously applied for phylotyping in our earlier study [10]. It consisted of 59 complete genomes whose phylogroups were previously identified in the original publications and 65 randomly selected chromosomes not assigned to phylogroups (listed in Supplementary Table S1 in [10]). Set 2 was assembled to validate phylotyping consistency. It was composed from 6300 completed bacterial genomes deposited to NCBI GenBank. After removing identical sequences and the 124 genomes from Set 1, the remaining genomes were randomly split into 10 subsets. Each subset was then back-supplemented with the 124 genomes from Set 1. Using the sets of non-redundant 18-mers from each genome and *E. albertii* KF1 chromosome as an outgroup, we constructed 10 draft phylogenetic trees via the neighbor-joining method based on pairwise Sørensen’s distance matrices [54]. Clusters corresponding to all eight phylogroups were identified in each subset, and genomes distinct from Set 1 and each other were selected and combined. After iterative phylogenetic refinement to minimize duplicates and overlaps with Set 1, 154 genomes were finalized for Set 2 (listed in Supplementary Table S1).

### 2.3. Barcoding of the E. coli Phylogroups

Barcoding and intraspecific taxonomic analysis were performed as previously described [51]. In brief, we used a local copy of the NCBI GenBank database (28 February 2023 release), containing 45,566 fully assembled bacterial genomes, 51,914 plasmid sequences and the human reference genome (GRCh38). Hash indices were generated for all non-redundant 18-mers across these sequences. For *E. coli* genomes (both sets), we excluded 18-mers present in any bacterial genome or plasmid in the local copy of the NCBI GenBank database (including *E. coli* plasmids), except for 3616 *E. coli* genomes and 233 genomes of closely related *Shigella*. The 18-mers from *Homo sapiens* chromosomes were also filtered out. Unique 18-mer barcodes for each phylogroup were obtained by stepwise removal of shared 18-mers across phylogroups. The distribution of model genomes among the eight phylogroups and their barcode sizes are summarized in Table 2.

The 18-mer barcode sets for all phylogroups are available in our GitHub repository (https://github.com/marsfro/ecoli_counter/ (accessed on 6 June 2025)). Hashing and barcoding were conducted as described in [10,51] using the 64-bit UniSeq software on a high-performance server (configuration: 2 Xeon Gold 5218, 64 GB RAM).

### 2.4. Alternative Intraspecific Phylotyping of E. coli

Alternative UniSeq-based phylotyping of Set 1 genomes was performed as described above, but with a modified *k*-mer selection approach. Instead of using unique marker *k*-mers absent in other bacterial genomes (one of the two UniSeq program options [10]), we used all non-redundant 18-mers present in each genome for clustering. This approach is functionally similar to MASH [15], differing from phylotyping based on unique barcodes [10,51]. Next, pairwise Sørensen similarity indices [54] were computed for the 124 sets of 18-mers. A phylogenetic tree was then reconstructed from the resulting distance matrix using the neighbor-joining method [55] in MEGA X [56].

### 2.5. Analysis of Human Metagenomes Based on Phylogroup-Specific Taxonomy

Phylogroup-specific *k*-mers were used to assess the relative abundance of each *E. coli* phylogroup in target metagenomes. Reads containing at least one phylogroup-specific *k*-mer were counted and assigned to their respective phylogroups. To enable comparison across phylogroups with differing barcode size (Table 2), read counts were normalized using the average barcode size (554,688 *k*-mers). A Python script kmers_ecoli_counter.py for identifying and counting phylogroup-specific reads in WGS metagenomes is available at https://github.com/marsfro/ecoli_counter/ (accessed on 6 June 2025). The resulting relative abundances are provided in Supplementary Table S2. To evaluate potential links between *E. coli* intraspecific diversity and interspecific relationships, we estimated the abundance of three dominant human gut genera (*Bacteroides*, *Prevotella* and *Ruminococcus*) characteristic of different enterotypes. Following standard pre-processing, taxonomic profiling was performed using Centrifuge v.1.0.4 software [57].

### 2.6. Machine Learning Methods for Binary Clustering

The normalized percentages of the eight *E. coli* phylogroups from control and pathological/treatment-derived samples served as features (variable Y) in machine learning models. Clustering was performed in Python (v. 3.10.9) using the Scikit-learn libraries (sklearn, v. 1.3.0) [58]. Datasets of PRJEB77747774 [48] and PRJEB33500 [52] with a large number of independent variables were partitioned into training (80%) and test sets (20%) via train_test_split. Three binary classification models were used: logistic regression (LR), random forests (RFs), and gradient boosting (GB). To optimize the hyperparameters of these models, we performed a grid search using the GridSearchCV function from sklearn. For LR, the regularization parameter C was tuned with values of 0.1, 1 and 10. The RF classifier was tuned with the number of estimators (n_estimators) set to 50, 100 and 200, and the maximum tree depth (max_depth) was set to 3, 4 and 5. For the GB classifier, n_estimators were set to 50, 100 and 200, and learning_rate to 0.1, 0.05 or 0.01. GridSearchCV systematically evaluated all parameter combinations for each model. The best parameters were selected based on 5-fold cross-validation and the area under the receiver operating characteristic curve (ROC-AUC) as the evaluation metric. The optimal values of hyperparameters are given in figure captions.

### 2.7. Dataset Visualization

Virtual visualization of clusters was performed using the Uniform Manifold Approximation and Projection (UMAP) technique for dimension reduction [59]. The relative abundances of the eight phylogroups were converted to a standard input format using the StandardScaler option from the sklearn.preprocessing module in Python. The fit_transform option was then applied to center the features around their mean and scale them to unit variance. Next, the UMAP technique was used to reduce dimensionality and visualize the datasets in a space with lower dimensionality. Finally, the image was created using UMAP CLASS option from the UMAP library (version 0.5.2) with the following hyperparameters: n_neighbors = 35 or 20 (number of neighboring data points to consider) and min_dist optimized in the range 0.1–1.0 (the effective minimum distance between embedded points). Due to UMAP’s stochastic optimization process, images generated with identical parameters may vary across runs. We therefore evaluated classification robustness by assessing cluster number consistency and the occurrence of merging/splitting events.

### 2.8. Statistical Analysis

For statistical analysis, numerical values corresponding to duplicated probes in the PRJEB33500 project and multiple samples from 7 to 15 donors in PRJEB28097 were averaged. The normality of data distribution across datasets was assessed using a one-sample *t*-test in SigmaPlot (v.11). As most datasets did not meet normality assumption, group comparisons were performed using the nonparametric Mann–Whitney–Wilcoxon test via the SigmaPlot Compare Two Groups option based on median differences [60]. Due to non-normality, variability was also assessed using mean absolute deviations (MADs) instead of two-way ANOVA. Sample-specific deviations from the mean were compared between datasets using a median-based test. Datasets exhibiting a statistically significant increase in variance (*p* < 0.05) and a greater interquartile range (IQR) than baseline (see figure legends for cutoffs) were classified as significantly diversified. We used the Pearson correlation coefficient (R) to evaluate intraspecific associations between different phylogroups and their interspecific connections with *Bacteroides*, *Prevotella* or *Ruminococcus* in all datasets. The statistical significance of R was estimated using a VassarStats online tool (http://vassarstats.net/tabs_r.html (accessed on 6 June 2025)) [61]. To check the robustness of statistical assessments, we performed jackknife resampling by iteratively excluding a single sample at a time from the original dataset [62]. This analysis identified 7 outliers out of 558 samples that disproportionately influenced the outcomes; these were excluded from subsequent analysis (see Supplementary Tables S2 and S3). For machine learning-derived data, statistical significance was assessed using Student’s *t*-test in Python.

## 3. Results

In our previous study [10], we performed *E. coli* phylotyping using a *k*-mer-based approach and MLST. For MLST, a combined set of 27 marker genes proposed in [4,63,64,65] was implemented. The individually aligned sequences of these genes were concatenated and a phylogenetic tree was constructed using the IQ-TREE [66]. For the *k*-mer-based approach, phylogenetic trees were inferred from a pairwise distance matrix of *Escherichia coli*/*Shigella*-specific 18- and 22-mers (124 genomes total), identified using the UniSeq algorithm [10]. Both methods produced topologically identical trees. Their clustering precisely matched ClermonTyping [6], though 14 strains showed discrepancies compared to the MASH-based clustering used in [11]. These included 12 strains classified as C, which in [11] were assigned to group B1, and 2 strains, classified in our study as B1 bacteria, were assigned to group C. Since the MASH algorithm, originally developed to assess intraspecific polymorphism in bacteria, estimates genomic distances based on “mutation rates” using representative 21-mers [15], the observed discrepancies between our classification and MASH-based typing might be due to difference in the marker *k*-mer sets, used for clustering. Given that the accuracy of taxonomic analysis critically depends on the quality of *k*-mer barcodes, we re-evaluated the phylotyping of our 124 genomes using the option of UniSeq software analogous to MASH [10,51].

### 3.1. Validation of Intraspecific E. coli Phylotyping Using MASH-like Option of UniSeq Pipeline

**Figure 1:**
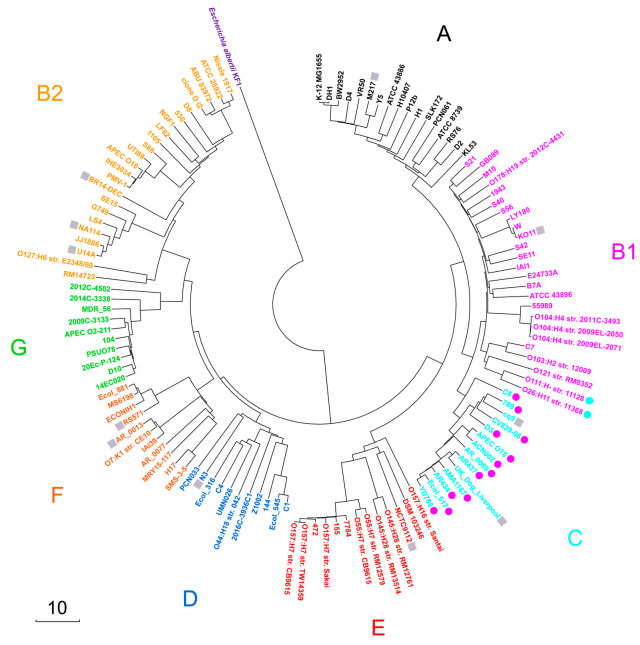
Phylogenetic tree of 124 *E. coli* strains constructed using the neighbor-joining method [55] in MEGA X [56] based on a pairwise distance matrix derived from sets of represetative 18-mers. The scale bar shows the Sørensen distance (percentage). The eight *E. coli* phylogroups are color-coded. A set of *Escherichia albertii* KF1-representative 18-mers was used as an outgroup. Strains with discordant phylotyping (B1 vs. C) relative to [11] are highlighted with colored circles, while strains not analyzed in [11] are denoted as gray squares.

Unlike our previous analysis, which used *k*-mers unique for *E. coli*/*Shigella*, this implementation considered all non-redundant 18-mers present in each genome. Then, pairwise Sørensen similarity indices [54] were computed for all 124 18-mer sets and the phylogenetic tree was constructed from the pairwise distance matrix using the neighbor-joining method [55]. As a result, the constructed tree (Figure 1) showed identical topology to our earlier phylogeny based on unique 18-mers (Figure 3 in [10]).

Thus, five distinct approaches, including MLST analysis using 27 marker genes [10], in silico PCR and MASH via ClermonTyping resource [6], and two UniSeq-based techniques ([10] and Figure 1), consistently yielded congruent phylotyping results. Thus, the same 124 genomes as in our previous study [10] were used for phylotyping with their distribution across phylogroups detailed in Table 2 (Set 1). Strains O111:H- str. 11128 and O26:H11 str. 11368 were assigned to phylogroup B1, while strains C8, 789, cq9, CV839-06, D3, APEC O78, ACN002, AR_0069, AR437, UK_dog_Liverpool, AM1167, AR434, Ecol_517 and YD786 were considered as members of phylogroup C.

### 3.2. Assessment of Barcoding Specificity

While both UniSeq- and MASH-based typing methods are equally effective for phylogenetic analysis, only UniSeq, which relies on unique marker *k*-mers absent in other bacterial genomes, enables intraspecific taxonomic analysis of natural microbial communities. To estimate the abundance of bacterial groups in metagenomes, the algorithm quantifies reads containing their specific 18-mer markers. The accuracy of these estimates depends on both the number and specificity of the 18-mer markers used. Increasing the number of known genomes within each phylogroup improves barcode specificity. However, expanding the reference database for filtering reduces the cumulative barcode size. Since natural microbiomes contain many bacteria not represented in the reference database, uncharacterized microorganisms will inevitably generate reads that are misassigned to known groups. To account for this bias, two complementary approaches were applied.

**Figure 2:**
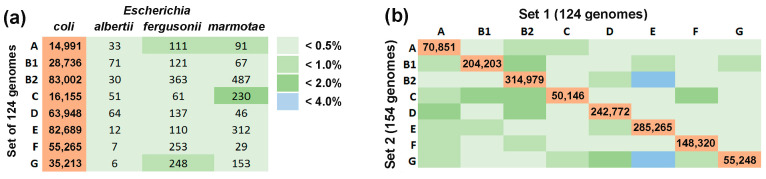
Intraspecific barcoding of *E. coli* using 124 genomes with verified phylogroup identity provided marker 18-mers that (**a**) had limited cross-species similarity and (**b**) were acceptable in terms of taxonomic analysis specificity. (**a**) Average number of marker 18-mers in 124 *E. coli* genomes (*coli*) compared to chromosomes of *E. albertii* (CP130156.1, CP141901.1, CP157789.1), *E. fergusonii* (CP099328.1, CP125351.1, CP137855.1) and *E. marmotae* (CP099344.1, CP099351.1, CP173213.1). The corresponding columns display the number of marker 18-mers found in each genome and averaged by species. The color code represents potential error by the relative abundance of those 18-mers in other *Escherichia* compared to their presence in *E. coli*. (**b**) Overlap of barcodes from the 124- and 154-genome sets, showing numbers of shared 18-mers within the same groups (diagonal) and unintended cross-phylogroup overlaps (shades of green and blue). Match percentages were assessed relative to the smaller barcode in each pair. A unified color code denotes overlaps in both panels.

To assess the species specificity of marker *k*-mers, we estimated their average number in the 124 genomes used for barcoding. These values were then compared with the *k*-mer content of other *Escherichia* species (*E. albertii*, *E. fergusonii* and *E. marmotae*) (Figure 2a). Genome selection was based on two criteria: (1) absence from the reference dataset of their close homologs to prevent 18-mer filtering during barcode construction, and (2) mutual evolutionary dissimilarity as determined by the phylogenetic tree topology (constructed similarly to Figure 1).

Three genomes per species appeared to be sufficiently representative with the highest observed overlap occurring between *E. coli* group C barcodes and 18-mers of *E. marmotae* RHB35-E2-C08 (1.4%). The average overlap across other combinations was significantly lower (0.34 ± 0.07%). These estimates suggest that the presence of uncharacterized *Escherichia* is unlikely to substantially affect the accuracy of *E. coli* phylogroup quantification in natural microbiomes. For the second trial, we constructed Set 2 containing 154 genomes with phylogroup identities assigned via ClermonTyping and the UniSeq approach (Table 2). These genomes were selected from the NCBI GenBank as described in the Materials and Methods Section and independently barcoded using the same reference database as for Set 1. Phylogenetic reconstruction confirmed clear separation of genomes into eight phylogroups with no discordance against expected identities (Supplementary Figure S1). To expand the training set, we increased the number of model genomes in all phylogroups except B1. This enlarged their barcodes by incorporating additional *E. coli*/*Shigella*-unique 18-mers, but also increased overlap between barcodes of different groups. Subsequent removal of similar sequences from phylogroup-specific barcodes reduced their size. Consequently, all groups except E exhibited a reduction in the number of marker 18-mers compared to Set 1 (Table 2).

We next quantified the overlap between the two sets for each phylogroup and revealed that 20–52% of 18-mers from the smaller set (usually Set 2) were shared with Set 1 barcodes. Overlaps between different groups ranged from 0.049% to 3.950% (Figure 2b) with the highest cross-phylogroup similarity observed between group E of Set 1 with groups B1 (2.89%) and G (3.95%) from Set 2. The average overlap across other phylogroup combinations was substantially lower (0.55 ± 0.08%), only marginally exceeding typical interspecific overlap levels (0.34 ± 0.07%) (Figure 2a,b). Therefore, we proceeded with Set 1 barcodes for all subsequent intraspecific taxonomic analyses.

### 3.3. Colorectal Adenoma and Carcinoma Had Different Impact on the Distribution of E. coli Phylogroups in the Intestinal Microflora

**Figure 3:**
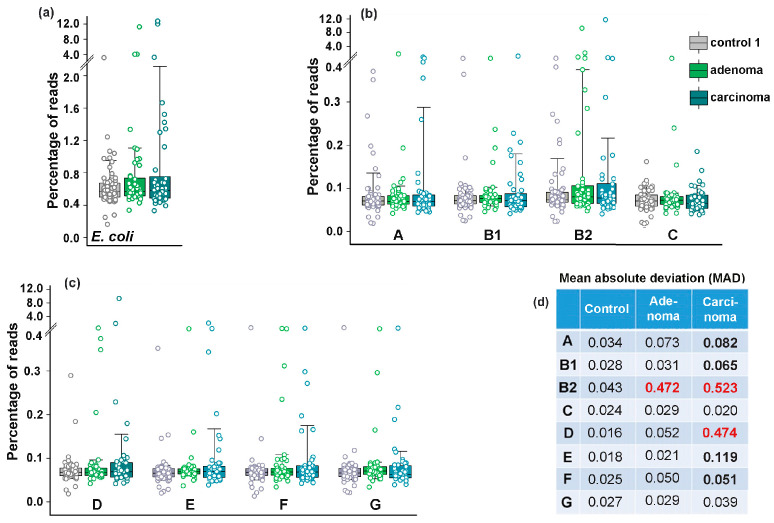
The diversity in abundance of *E. coli* (**a**) and its phylogroups (**b**,**c**) increased in the microbiomes associated with colon diseases (**d**). The box plots show the percentages of reads with phylogroup-specific 18-mers (**b**,**c**) or *E. coli*/*Shigella*-specific 18-mers (**a**). Significance of MAD alterations was assessed using Mann–Whitney–Wilcoxon test [60] and interquartile interval range (IQR). MADs with statistically significant changes (*p* < 0.001) and IQRs increased by at least 1.5 times are shown in bold. An increase in IQR by more than 2 times is indicated in red.

Consistent with previous reports [35], we anticipated that at least carcinoma would either promote greater persistence of phylogroup B2 in the gut or otherwise affect *E. coli* phylogroup homeostasis. The average abundance of this phylogroup was indeed higher in the samples obtained from patients with adenoma and carcinoma (0.349% and 0.362, respectively) than in the control group (0.097%) (Supplementary Table S2, Figure 3). 

Only group D bacteria increased their presence to almost the same level in microbiomes associated with carcinoma (Supplementary Table S2), but all changes, along with those in total *E. coli* abundance, were not statistically significant (Figure 3a–c). Nevertheless, we observed an a priori unexpected increase in the variability of *E. coli* phylogroup abundance across different biological samples. In particular, adenoma increased the mean absolute deviation (MAD) in the presence of B2 bacteria, while carcinoma promoted significant variability for six phylogroups, with the greatest impact on B2 and D group bacteria (Figure 3d).

**Figure 4:**
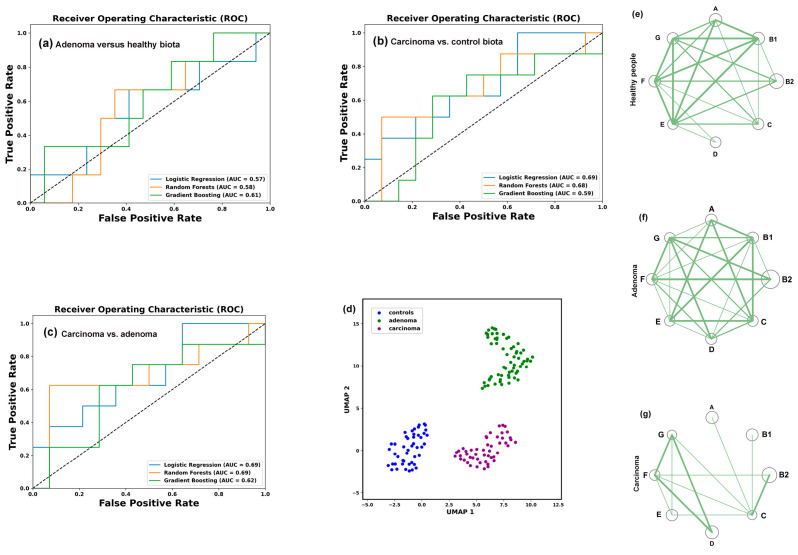
While binary clustering failed to differentiate *E. coli* phylogroup composition between control samples and adenoma-associated microbiomes (**a**), RF and LR models (**b**,**c**), UMAP dimensionality reduction algorithm [59] (**d**) and intraspecific correlations (**e**–**g**) effectively detected changes in *E. coli* homeostasis. The C parameters used for binary classification with LR were 0.1 (**a**) and 1.0 (**b**,**c**). The parameters for clustering with RF were max_depth = 5, n_estimator 200 (**a**,**b**) and 50 (**c**). The best learning_rate/n_estimator combinations for GB were 0.1/100 (**a**), 0.05/200 (**b**) and 0.1/200 (**c**). UMAP clustering (**d**) was performed using parameters n_neighbors = 35, min_dist = 0.7. (**e**–**g**) Network visualization of intraspecific correlations among *E. coli* phylogroups based on Pearson’s correlation coefficient (R). Node size reflects mean phylogroup abundance in samples. Statistically significant correlations are indicated by lines with thickness reflecting their strength: *p* < 0.00001 (thick), *p* < 0.001 (medium), *p* < 0.05 (thin).

Assuming that the difference in *E. coli* homeostasis between the usually harmless adenoma and the dangerous carcinoma reflects variable ways in which *E. coli* adapts to the specific environment created in the gut, we used machine learning algorithms to assess whether the frequency profile of phylogroups can distinguish between a healthy and pathological state. Normalized percentages of the eight phylogroups in control and pathological conditions were used as features defining the target variable Y in machine learning models for each type of samples. Binary classification was performed using logistic regression (LR), random forest (RF), and gradient boosting (GB) models (Figure 4a–c). The best hyperparameters for each model were obtained as described in the Materials and Methods Section. Their performance was evaluated using the area under the curve (AUC) as a distance-based metric for the receiver operating characteristic (ROC) curves.

The resulting ROC-AUC scores of 0.57 (LR), 0.58 (RF) and 0.61 (GB) were close to 0.5, indicating a random distribution of the control samples and samples from patients with colorectal adenoma (Figure 4a). However, when the control set was compared with the carcinoma patient samples (Figure 4b), the AUC scores were higher and, at least for the LR model, separated the two sets with 73% accuracy. The discriminatory power of this model also distinguished between *E. coli* populations in the microbiomes of people with colorectal carcinoma and adenoma with approximately the same accuracy (Figure 4c). An algorithm based on nonlinear dimensionality reduction (Uniform Manifold Approximation and Projection, UMAP [59]) clearly separated all three sets of biological samples (Figure 4d), reflecting distinct phylogroup compositions.

Based on this observation, we next evaluated intraspecific correlations in the abundance of *E. coli* phylogroups within the microbiomes (Supplementary Table S3). In the control set (Figure 4e) and in the microbiota associated with adenoma (Figure 4f) all phylogroups exhibited positive correlation with each other. Although not all correlations were statistically significant, this likely suggests that bacteria from different groups do not specialize in entirely independent functions within *E. coli* populations. The number of correlated groups in samples obtained from the microbiomes of patients with carcinoma, on the contrary, was much smaller, and the B2 group showed non-relationships with A, B1, D and E phylogroups (Figure 4g). Thus, while the abundance of *E. coli* phylogroups did not differ significantly among microbiomes adapted to different host physiological states (Figure 3b,c), their interaction patterns appeared sensitive to the chronic alterations induced by carcinoma.

### 3.4. Bimodal Response of E. coli to Antibiotics and Recovery with Probiotics

**Figure 5:**
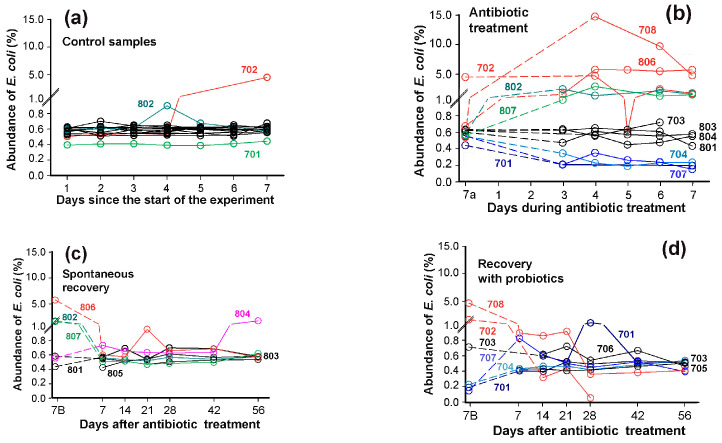
Dynamics of *E. coli* abundance (sum of all phylogroups) in healthy human microbiomes. (**a**) Stable baseline levels during 7-day pretreatment period. (**b**) Differential response patterns following antibiotic administration. (**c**,**d**) Recovery patterns in the absence (**c**) or presence (**d**) of probiotic supplementation. Numerals indicate sample IDs (Supplementary Table S2). Left time points in (**b**–**d**) represent endpoints of preceding stages. Whenever possible, dashed lines connect longitudinal measurements from the same microbiome. Samples of colored plots are discussed in the text.

Despite being inherently susceptible to most clinical antimicrobials, *E. coli* has a remarkable capacity to acquire resistance genes through horizontal gene transfer [40,67]. They are distributed across all phylogroups and may function collectively to counteract antimicrobial drags. Using data from the PRJEB28097 project [49,50] we aimed to characterize the individual responses of *E. coli* groups to ciprofloxacin, resistance to which was observed among isolates of all phylotypes [43,68,69]. The selected dataset contained stool samples from healthy volunteers receiving ciprofloxacin (500 mkg, twice daily) and metronidazole (500 mkg, three times daily) [49,50]. As metronidazole exhibits activity against *E. coli* only in the presence of other susceptible bacteria [70], this antibiotic combination enabled detection of community-dependent response. While the abundance of *E. coli* in these microbiomes was comparable to that observed in the PRJEB7774 project (Figure 2a and Figure 5a), this set allowed for longitudinal tracking of antibiotic-induced microbiome alterations (Figure 5b).

Prior to antibiotic administration, *E. coli* abundance was stable in 13 out of 15 microbiomes (Figure 5a). Two exceptions were observed: sample 802 showed only a transient increase, while in sample 702, *E. coli* exhibited significant spontaneous expansion beginning on day 5 of the pre-testing phase. This elevated level persisted until day 5 of antibiotic treatment, when a temporarily decline occurred (Figure 5b). As expected, antibiotic exposure caused divergent effects on *E. coli* abundance across individuals. Among the twelve samples analyzed dynamically, four (703, 801, 803, 804) demonstrated complete antibiotic insensitivity; microbiomes from samples 702, 708, 802, 806 and 807 showed significant *E. coli* proliferation, while samples 701, 704 and 707 displayed the opposite pattern, with notable *E. coli* reduction (Figure 5b).

Following seven days of spontaneous recovery, most microbiomes returned to near-baseline *E. coli* levels. Only sample 804, previously identified as antibiotic-insensitive, displayed unexpected *E. coli* expansion at the end of experiment (Figure 5c). Spontaneous recovery of *E. coli* in microbiomes took less than 7 days (Figure 5c). However, supplementation with probiotic mixture [49,50] extended this period: even after 3 weeks, *E. coli* abundance still differed from baseline levels and remained unstable in several samples (Figure 5d). This delayed recovery aligns with the slower and incomplete reconstitution of mucosal microbiomes observed for the same biological samples [49]. Thus, the abundance of *E. coli* in fecal microbiomes is sensitive to the presence of both antibiotics and probiotics.

### 3.5. The Response of E. coli to Antibiotics Was Not Uniform Among Phylogroups

Observing the absence of a strong time dependence in *E. coli* abundance for most samples from the same donor within a given experimental stage (Figure 5), we assessed this dependence for each phylogroup using the mean and standard deviation as parameters. Among the control set, 101 of 120 dynamic profiles exhibited StDs below 10% of the mean. However, the *E. coli* outbreak in donor’s 702 biota on day 7 during the pre-treatment period (Figure 5d), along with elevated average values, increased the StDs across all groups to over 50% of the mean values and reached 196% for group B1. Consequently, this sample (ID ERR2750008) was excluded from the analysis (Supplementary Table S2). In antibiotic-treated samples, 79% of profiles had StDs ranging from 5 to 40%, whereas 44 of 56 profiles from spontaneously recovered microbiomes had StDs less than 15% of the mean. The instability of *E. coli* persistence in donor 702 samples during probiotic-assisted recovery (Figure 5d) affected all eight phylogroups, resulting in StDs ranging from 61 to 86% of the mean. In contrast, 46 of the remaining 56 samples had StDs below 30%. Nevertheless, the mean phylogroup abundances in spontaneously recovering samples from donor 702 were broadly consistent with those of other donors, so none of these samples were excluded. Overall, the variability in phylogroup abundance across samples at each stage typically fell within the expected range for routine biological experiment. This allowed us to use the mean values for each microbiome at each stage as independent variables for statistical assessment.

**Figure 6:**
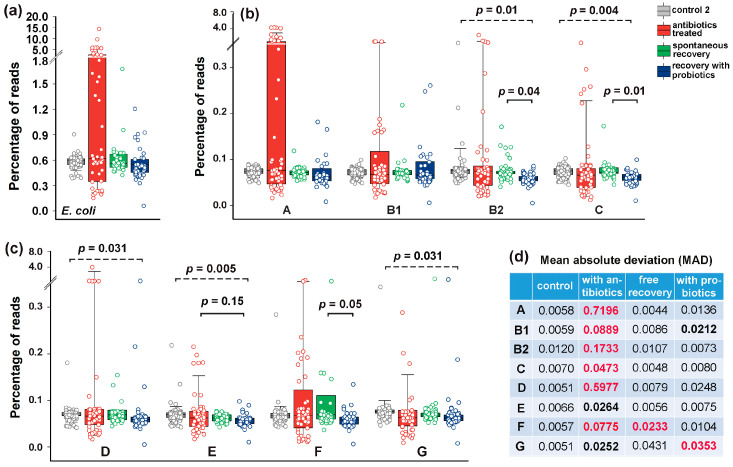
Phylogroup-dependent response of *E. coli* to antibiotic and probiotic supplementation. Scatter plots show the percentages of reads containing (**a**) *E. coli*-specific or (**b**,**c**) phylogroup-specific 18-mers. (**b**,**c**) Symbols show percentage of phylogroups in microbiomes, while box plots display their mean values averaged across samples from individual donors. (**d**) Statistical significance of MAD alterations was assessed using Mann–Whitney–Wilcoxon test [60] and interquartile interval range (IQR). MADs with IQR increase of more than 3-fold and statistically significant changes (*p* < 0.05 vs. controls) are bolded. Changes with *p* ≤ 0.001 are highlighted in red.

The bidirectional response of *E. coli* to antibiotics (Figure 5b and Figure 6a) significantly increased the MAD of all phylogroups without statistically significant changes in their abundance (Figure 6b–d). The main contribution to the variability of *E. coli* was made by group A (Figure 6a,b), the variance of which was less pronounced in microbiomes associated with colon diseases (Figure 3d). Consistent with the dynamic plots (Figure 5c), one week of spontaneous recovery following antibiotic treatment significantly reduced *E. coli* variance and abundance to near-baseline levels (Figure 6d).

However, this effect was not similar for all groups. Phylogroup F, in particular, retained significantly higher variability compared to controls (Figure 6c,d). The influence of probiotics tested on an independent group of volunteers was also apparent: most groups exhibited reduced persistence in microbiomes, though group G remained variable throughout the extended recovery period (Figure 6c,d). Therefore, the response of *E. coli* to antibiotics and probiotics was not uniform for all of its phylogroups.

### 3.6. Post-Treatment Recovery Partially Restored the Intra- and Interspecies Balance Disrupted by Antibiotics, but Not the Original Correlation Between Phylogroups and the UMAP Cluster

**Figure 7:**
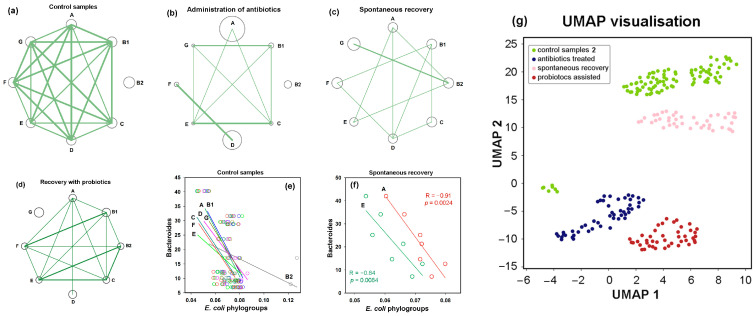
Networks of intraspecific *E. coli* correlations and their connections with other taxa. (**a**–**d**) Network visualization of intraspecific correlations among *E. coli* phylogroups, which were disrupted by antibiotics (**b**) and partly restored in a probiotic-dependent manner (**c**,**d**). Node size represents mean phylogroup abundance across samples. Lines depict statistically significant correlations, with thickness reflecting their strength: *p* ≤ 0.00001 (thick), *p* < 0.001 (medium), *p* < 0.05 (thin). (**e**,**f**) A trend toward negative interspecific correlations with *Bacteroides* in control samples (**e**) became statistically significant for two phylogroups during spontaneous recovery (**f**). (**g**) UMAP clustering (parameters: n_neighbors = 35, min_dist = 1.0) visualizes differences in *E. coli* homeostasis among four gut microbiota states.

In control microbiomes, all *E. coli* phylogroups were present at nearly equal proportions, and their percentages were highly correlated (Figure 7a). Only group B2 showed no statistically significant intraspecific associations (Supplementary Table S3). By inducing divergent changes in all groups, antibiotics disrupted this equilibrium. Consequently, more than half of the intraspecific correlations were lost (Figure 7b). Within eight weeks after antibiotic exposure, the relative abundance of all phylogroups had nearly returned to baseline levels (circles in Figure 7a,c). This recovery was particularly evident for groups A and D, which had expanded during antibiotic treatment, as well as for the antibiotic-suppressed groups B1, C, E, F and G, which rebounded close to their original abundances. However, the number of intraspecific links remained reduced compared to control samples, and the network structure differed from both baseline and antibiotic-perturbed states. For instance, group B2 bacteria, which showed no significant correlations with other groups in control and antibiotic-treated samples, developed significant connections with groups A, E and G (Figure 7c). Probiotic-assisted restoration, which suppressed the expansion of groups A and D, established statistically significant associations between B2 bacteria and groups B1, C, E and F, but disrupted all intraspecific links of group G, which “survived” antibiotic exposure. Notably, supplementation of recovering microbiomes with probiotic mixture resulted in a statistically significant reduction in the abundance of phylogroups B2, C and F without essential impact on other groups (Figure 6b,c). This observation aligns with previous findings showing that administration of *Bifidobacterium longum* and *Lacticaseibacillus paracasei* suppressed *Escherichia* in the gut microbiomes of laboratory rats, though the effect was observed only in one enterotype [71]. Together, these results suggest that *E. coli*’s response to exogenous bacteria depends on interspecific microbial interactions.

To investigate this, we estimated the abundance of dominant enterotype-associated genera (*Bacteroides*, *Prevotella* and *Ruminococcus*) [1] in all samples using the metagenomic classifier Centrifuge [57]. In control samples, we observed consistent negative correlations between all *E. coli* phylogroups and *Bacteroides* with statistically significant associations for groups A, B1, C, D and G in the range −0.56 < R < −0.44 (Figure 7e).

*Prevotella*, on the contrary, positively correlated with all *E. coli* phylogroups (significant associations for groups A, B1 and D in the range 0.52 < R < 0.61), while associations with *Ruminococcus* were insignificant (Supplementary Table S3). Antibiotics preserved the weak negative correlations between *Bacteroides* and three *E. coli* phylogroups but inverted the relationship with group B2 (R = 0.40 ± 0.04 in jackknife analysis). During spontaneous recovery, *Bacteroides* re-established their negative link with B2 and strengthened negative associations with groups A and E (Figure 7f). In the presence of probiotics, only group G maintained its original connections with *Bacteroides*, while all other groups showed an inverse trend (Supplementary Table S3).

Antibiotics converted the positive correlations between *E. coli* groups and *Prevotella* into weak negative connections, while simultaneously establishing positive links with *Ruminococcus*. During spontaneous recovery, *Prevotella* re-established positive links with groups A, B2, E and G, whereas probiotics restored positive associations only with groups B2 and C. Interactions with *Ruminococcus* spontaneously reverted to negative correlations for all *E. coli* phylogroups. However, probiotic intervention not only re-established their positive links with group B2 but also created a new positive association with group C.

Therefore, although interspecific connections showed lower R-values than intraspecific ones, we detected multiple instances of their significant alterations in response to different interventions (Supplementry Table S3). These rearrangements sometimes reversed the correlation type, which was never observed for intraspecific connections. While most phylogroups followed similar association patterns with dominant taxa (positive with *Prevotella* or negative with *Bacteroides*), some established individual interspecific links. For example, only group G negatively correlated with *Bacteroides* upon probiotic therapy and groups B1, C, E and G exhibited unusual positive correlations with *Ruminococcus* under antibiotic treatment. These distinctive connections may play a crucial role in shaping intraspecific linkage networks (Figure 7a–d), and creating specific homeostasis within the *E. coli* population (Figure 7g).

### 3.7. Idealizing E. coli Intraspecific Balance, the Mediterranean Diet Intensified Its Negative Link to Bacteroides and Unlocked Bidirectional Connections with Prevotella

**Figure 8:**
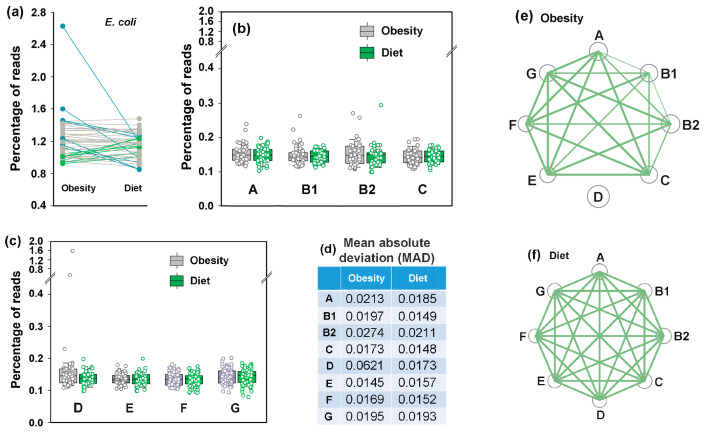
The Mediterranean diet did not significantly alter the overall abundance of *E. coli* or its phylogroups in the gut microbiomes, but improved intraspecific balance without induced diversification. (**a**–**d**) Symbols in line and box plots display the percentages of reads containing either all *E. coli*-specific 18-mers (**a**) or phylogroup-specific 18-mers (**b**,**c**) in the metagenomes. Plots in (**a**) are color-coded to indicate ≥10% increase (green) or decrease (blue) from baseline. Changes within 10% of baseline are shown in gray. (**d**) Mean absolute deviations estimated from the mean values of 43 paired samples. (**e**,**f**) Network visualization of intraspecific correlations among *E. coli* phylogroups before and after MD. Node sizes correspond to mean phylogroup abundances, while the connecting lines represent statistically significant correlations, with thickness reflecting their strength: *p* ≤ 0.00001 (thick), *p* < 0.001 (medium), *p* < 0.05 (thin).

The PRJEB33500 project dataset [52] comprises duplicate fecal samples from 43 overweight/obese volunteers collected before and after an 8-week Mediterranean diet (MD) intervention. Both intra- and interspecific *E. coli* relationships were assessed using the mean values of duplicate samples (Figure 8). The average abundance of *E. coli* in the microbiomes of overweight individuals (Figure 8a) was higher than in healthy donors from the other two analyzed projects (Figure 3a and Figure 6a). Although the adaptive response of microbiomes to dietary restriction was also bidirectional (Figure 8a–c), the divergence was much less pronounced than that observed under antibiotic exposure. Only six microbiomes exhibited moderate *E. coli* persistence increases (10–30% relative to baseline) (Figure 8a), while seven samples showed abundance reduction (10–55%), including two microbiomes in which high *E. coli* levels were predominantly associated with phylogroup D outbreaks (Figure 8c).

Notably, dietary restriction led to a reduction in MAD (Figure 8d), possibly reflecting the stabilizing effect of a balanced diet. While no significant changes were observed in the average abundance of *E. coli* phylogroups (Figure 8b,c), intraspecific balance improved markedly. The balanced presence of all phylogroups (nodes in Figure 8f) with the reduced abundance of phylogroup D bacteria in microbiomes with their initial excess (Figure 8c) resulted in strong correlations among all groups (Figure 8f). Thus, the Mediterranean diet may be proposed as a way to restore intraspecific homeostasis of *E. coli*.

**Figure 9:**
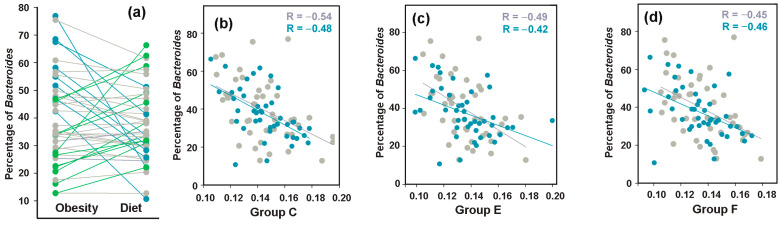
Bidirectional changes in *Bacteroides* abundance induced by Mediterranean diet adherence retained negative correlations with *E. coli* phylogroups. (**a**) Changes in *Bacteroides* prevalence were calculated from mean percentages in replicate fecal samples collected from 43 overweight individuals before and after 8 weeks of a diet. Plots are color-coded to indicate ≥10% increase (green) or decrease (blue) from baseline. Changes within 10% of baseline are shown in gray. (**b**–**d**) Representative negative correlation patterns between *Bacteroides* and *E. coli* phylogroups C (**b**), E (**c**) and F (**d**). Baseline and post-intervention samples are shown in gray and blue, respectively.

Based on previous studies, which demonstrated significant underrepresentation of *Bacteroides* in the gut microbiomes of obese individuals [72], we anticipated the detection of a difference in their presence in response to MD or resulting changes in their relationships with *E. coli*. The expected increase of at least 10% in *Bacteroides* abundance relative to baseline was indeed observed in 18 out of 43 model microbiomes, but in 14 samples, the percentage of *Bacteroides* decreased (Figure 9a). Therefore, even dominant bacterial genera employ bimodal adaptive responses to dietary restriction.

Prior to dietary intervention, only *E. coli* groups C, E, F (Figure 9b–d) and B1 (Supplementary Table S3) exhibited statistically significant negative links with *Bacteroides* (0.00044 ≤ *p* ≤ 0.036), but following 8 weeks of diet, such relationships became significant for all *E. coli* phylogroups (Supplementary Table S3). This connection aligns with observations from the PRJEB28097 project datasets (Figure 7e,f) suggesting that the antagonistic interaction with *Bacteroides* may represent a fundamental property of *E. coli*.

**Figure 10:**
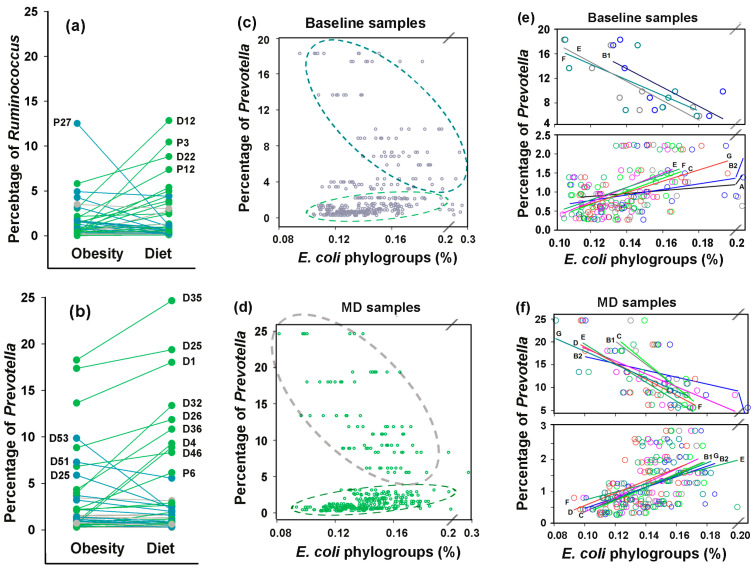
While inducing bidirectional changes in *Ruminococcus* and *Prevotella* abundance, MD did not significantly alter their presence in the gut, but revealed a bimodal relationship between the abundance of *E. coli* and the persistence of *Prevotella*. (**a**,**b**) Changes in *Ruminococcus* (**a**) and *Prevatella* prevalence were calculated from mean percentages in replicate fecal samples collected from 43 overweight individuals before and after 8 weeks of a diet. Plots are color-coded to indicate ≥10% increase (green) or decrease (blue) from baseline. (**c**–**f**) Scatter plots showing either the entire set of samples (**c**,**d**), or samples divided into two categories based on the percentage of *Prevotella* in the microbiomes (**e**,**f**). The color of the circles corresponds to the color of the regression lines of the eight groups. Ovals outline symbols with different correlation modes.

While the plant-based diet increased *Ruminococcus* abundance in 25 of 43 samples, it had little effect on the average percentage of bacteria from this genus (Figure 10a). Furthermore, we found no robust evidence of interspecific associations between *Ruminococcus* and *E. coli* (Supplementary Table S3). However, positive correlation between *E. coli* and *Prevotella*, anticipated based on their symbiotic growth in binary bacterial consortia [73], and the relationships found in the PRJEB28097 project datasets (Supplementary Table S3) emerged in an unexpected manner (Figure 10c–f). As a dominant genus in the second human enterotype [1], *Prevotella* was detected in all microbiomes, with a mean abundance approximately twice as high as that of *Ruminococcus* (Figure 10a,b). The high-fruit/vegetable, low-meat Mediterranean diet is beneficial for *Prevotella*-consuming complex carbohydrates and resulted in bidirectional adaptive changes in the persistence of this genus (Figure 10b). As a result, its weak positive correlations across the entire baseline dataset with groups C (R = 0.31, *p* = 0.021) and D (R = 0.46, *p* = 0.0009) disappeared (Supplementary Table S3). However, the scatter plots between abundances of *Prevotella* and *E. coli* phylogroups (Figure 10c,d) were far from both correlative and random, suggesting a potential bimodal relationship governing *E. coli* phylogroup abundance as a function of *Prevotella* levels. When the control set containing samples from overweight individuals was divided into two subsets (Figure 10e), the estimated R-values for correlation in the eight *Prevotella*-rich microbiomes (>5%) ranged from –0.53 to –0.84, with significant negative relationships observed for groups B1, D, E and F (0.009 < *p* ≤ 0.050). In contrast, significant positive correlations were detected in the 30 samples with low *Prevotella* abundance (<3%) for groups A, B2, C, E, F and G (0.42 < R < 0.54, 0.0019 < *p* ≤ 0.022). Thus, at least groups E and F may employ distinct communication modes with *Prevotella*.

Following dietary intervention, when intraspecific homeostasis became balanced (Figure 8f), the 11 *Prevotella*-rich microbiomes exhibited R-values ranging from −0.77 to −0.48 with statistically significant negative correlations in all groups except A (0.003 < *p* ≤ 0.019, Figure 10f), whereas all groups in 31 *Prevotella*-low samples demonstrated significant positive correlations (Supplementary Table S3). These findings are among the most important in our study, as they demonstrate the adaptive capacity of *E. coli* to employ divergent ecological strategies to optimize fitness depending on *Prevotella* abundance.

### 3.8. Assessing Difference Between Samples, Machine Learning Approaches May Also Be Implemented to Reveal Individual Similarity

**Figure 11:**
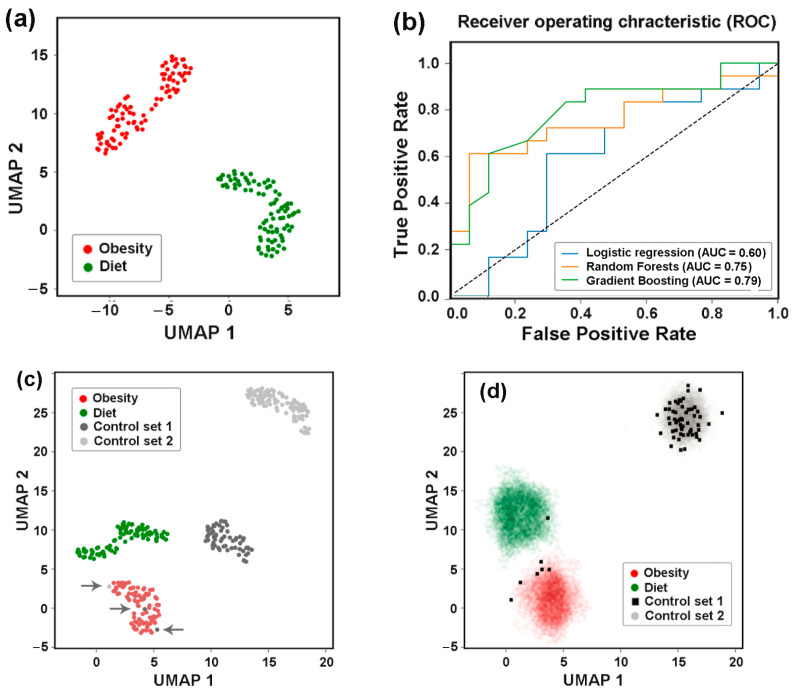
Visualization and statistical assessment of differences between *E. coli* populations (**a**,**b**) and optimization of UMAP for individual identity testing (**c**,**d**). (**a**) UMAP clustering of all 172 samples from the PRJEB33500 project (parameters: n_neighbors = 35, min_dist = 0.7). (**b**) ROC curves and AUC values for the same dataset, calculated using mean values of paired samples. (**c**) Joint UMAP clustering of Control Sets 1 and 2 (baseline samples from PRJEB7774 and PRJEB28097) with PRJEB33500 project samples. Arrows highlight samples with altered clustering in the combined classification. (**d**) Superimposed clusters from 65 images obtained in a “virtual diagnostics” experiment, when each sample from Control Set 1 (black squares) was individually added to a combined set of 264 samples from three other datasets. Clustering parameters for (**c**,**d**) n_neighbors = 20, min_dist = 1.

UMAP clustering clearly segregated pre- and post-MD microbiome samples into two distinct clusters (Figure 11a) and binary classification methods using at least two models (RF and GB) reliably distinguished between them (Figure 11b). However, when these two sets were jointly classified with control samples from the PRJEB7774 and PRJEB28097 projects, we observed four distinct clusters with the two control sets appearing perfectly separated (Figure 11c). It was not surprising that both sets of the dietary project differed from the control sets of PRJEB7774 and PRJEB28097, as the PRJEB33500 samples were obtained from apparently healthy individuals with a clear physiological peculiarity. The observed discrepancy between the two control sets from healthy volunteers (Figure 11c) was less expected and concerning. This divergence most likely represents a batch effect—a phenomenon when non-biological experimental factors introduce artefactual changes in the data. Known contributors to batch effects include differences in laboratory conditions, reagents, and sequencing instrumentation [74,75]. As the PRJEB7774 and PRJEB28097 samples originated from different sources (Beijing Genome Institute and Weizmann Institute of Science, respectively), their national/geographical characteristics may have driven their separation more strongly than the shared *E. coli* phylogroup distributions could enable their combination. However, the most important observation made in this part of the study was the presence of three control samples located outside their primary clusters (indicated by arrows in Figure 11c), suggesting their similarity to the samples of overweight people.

To evaluate, how samples from Control Set 1 would be distributed across the other three clusters when added individually, we performed a “virtual diagnostic” experiment (Figure 11d). This ignored the influence of Control Set 1′s specific features, while emphasizing the intraspecific similarities inherent in “healthy microbiomes”. The joint classification was performed 65 times with 265 samples, of which 92 belonged to Control Set 2, 172 were from dietary experiment sets, and 1 test sample was from Control Set 1 in each iteration. Because each substitution altered the UMAP, we only overlaid clusters rather than entire images (Figure 11d). Due to variations in cluster configurations in the individual UMAP images, the overlapping regions appeared as diffuse clouds. Black squares mark the locations of all Control Set 1 samples in the UMAP images. The majority (89.2%) of them clustered with samples obtained from healthy donors who had not received antibiotics or probiotics prior to sampling (Control Set 2). Six samples grouped with microbiomes from overweight individuals, while one joined to samples from Mediterranean diet followers. Even if six healthy donors from Control Set 1 were not clinically obese and one did not prefer a plant-rich diet, this non-random distribution of Control Set 1 samples among health-associated categories demonstrated the predictive power of intraspecific *E. coli* characteristics. Therefore, the distribution patterns of *E. coli* phylogroups or the intraspecific homeostasis of some other gut bacteria may reflect host physiological state, potentially serving as a basis for diagnostic applications.

## 4. Discussion

Based on the assumption that the genetic background establishing epistatic interactions with horizontally acquired genes also shapes phylogroup homeostasis of *E. coli*, which in turn governs both intraspecific and interspecific relationships within microbiomes, we investigated how *E. coli* phylogroup distribution in the human gut correlates with host physiological state. Our study addressed two key questions: (1) Are there any phylogroup-specific responses of *E. coli* to environmental changes caused by either chronic intestinal disorders or acute temporary disturbances? (2) How sensitive is the intraspecific balance of *E. coli* to such disruptions? While seemingly straightforward, these questions led us to discover unexpectedly profound rearrangements in both intraspecific relationships and in interspecific connections.

When assessing differences in the abundance of *E. coli* phylogroups between the control samples and under different physiological conditions, we observed a statistically significant decrease only in phylogroups B2, C and F following probiotic-mediated recovery after antibiotic treatment (Figure 6b,c). In most other cases, changes in mean abundance were not statistically significant. However, a phylogroup-specific response was evident in their variability. Even in microbiomes adapted to chronical colon diseases, phylogroups B2 and D exhibited a significant increase in mean absolute deviation (Figure 3d). More pronounced individual changes were observed in response to antibiotic treatment, with groups A, B2 and D contributing most to the adaptive variability of *E. coli* (Figure 6b–d).

Bidirectional changes in isogenic bacterial populations have long been recognized as “bistability” [76] or “bimodality” [77]. This phenomenon enables bacteria to adopt alternative survival strategies in adverse environments [76,77] or enhance virulence [78]. A classic example of such diversification is the emergence of persisters, i.e., subpopulations of cells that develop resistance to toxic agents or achieve antimicrobial tolerance by entering a dormant, slow-growing state [79]. Several mechanisms drive population bifurcation, including structural rearrangements and mutations in the genome [80], epigenetic modifications [78] and “transcriptional noise” [81,82], which, due to stochastic gene expression and feedback regulation [77], can cause segregation into two or more subpopulations. Some of the most compelling evidence for behavioral variability comes from single-cell studies [83,84]. Particularly relevant to our work are findings on species variability in natural microbiomes. For instance, using a mouse model of chronic colonization, W. Elhenawy and coauthors showed that Crohn’s disease-associated adherent-invasive *E. coli* (AIEC) isolates undergo host-specific adaptive diversification [85]. The authors identified two lineages that outcompeted the ancestral strain by enhancing invasion or improving acetate utilization in the gut. Although AIEC bacteria are distributed across all *E. coli* phylogroups, they are predominantly associated with group B2 [86], which displayed significantly increased variability in the gut microbiota of patients with chronic colorectal disorders and following antibiotic exposure (Figure 3d and Figure 6d). Consequently, phylogroups B2 and D formed the fewest intraspecific connections in the analyzed datasets (Figure 4e–g, Figure 7b–e and Figure 8e,f).

Phylogroup E, on the contrary, demonstrated the highest stability in baseline samples (0.008 ≤ MAD ≤ 0.0145) and along with the groups C and F formed the most extensive network of intraspecific correlations. By incorporating over 1000 alien genes [27,28] into the chromosomes of *E. coli* serotype O157:H7 alone [22] and domesticating more than 460 prophages [23], group E bacteria are forced to control expression of a larger number of genes than bacteria with smaller genomes of other groups. Thus, a weaker correlation between them was intuitively expected. Yet our analysis revealed the opposite pattern. It is therefore possible that the genetic background of group E bacteria, evolutionarily tuned to integrate alien genes, was also tuned to maintain the balance of *E. coli* phylogroups.

In evaluating interspecific relationships between *E. coli* phylogroups and dominant enterotype taxa, we observed predominantly negative correlations with *Bacteroides* (Figure 7 and Figure 9 and Supplementary Table S3). This is in line with several publications describing competitive relationships between these genera [87,88,89]. Following complete disruption of these links by antibiotics, the negative correlations with *Bacteroides* were spontaneously restored by group A bacteria and increased from an insignificant level for group E bacteria. By the end of the experiment, the interspecies connectivity network with *Bacteroides* had nearly returned to baseline levels. However, probiotic-mediated restoration converted phylogroups A and D links with *Bacteroides* from significantly negative to weakly positive (R = 0.46, *p* = 0.12). That means that the type of interspecific relations between *E. coli* phylogroups and dominant gut taxa may change depending on the presence of probiotic bacteria, which are only minor components in the human gut biota.

Interspecific interactions with *Ruminococcus* were mostly non-significant. Surprisingly, however, significant positive correlations were observed with phylogroups B1, C, E and G under antibiotic exposure, i.e., a condition when most microbial connections were disrupted (Supplementary Table S3). Interestingly, also, groups B1, C, and G formed similar associations in the microbiomes of overweight individuals with high *Prevotella* levels. After adherence to the Mediterranean diet, positive associations with *Ruminococcus* expanded to all groups, though statistically significant links shifted to groups D and E.

An even more unexpected observation emerged from our analysis of interspecific relationships between *E. coli* phylogroups and *Prevotella*. While the MetaHIT consortium’s human gut microbiome analysis predicted negative correlations between these taxa [1], our evaluation of the PRJEB28097 project control dataset revealed positive correlations between *Prevotella* and three *E. coli* groups (A, B1 and D). The same links persisted in samples from overweight individuals with low *Prevotella* abundance (positive correlation with all *E. coli* phylogroups except B1). Only when *Prevotella* abundance exceeded 5%, we observed the predicted negative correlations with groups B1, E and F (Figure 10e). Following dietary restriction, negative associations in *Prevotella*-rich biota were displayed in all phylogroups except A, while positive correlations in *Prevotella*-depleted microbiomes were enhanced and maintained by all phylogroups (Supplementary Table S3). Thus, both interaction types became significantly stronger and cannot be ignored. To our knowledge, this represents the first documented evidence of *E. coli* phylogroups switching between interspecific correlation types based on dominant taxa abundance. Unfortunately, we were unable to validate this phenomenon using the other analyzed datasets. In the colorectal disease project (PRJEB774), *Prevotella* abundance in all samples was very low (<1.2%) and no significant correlations with *E. coli* were observed, while the small number of independent variables in the PRJEB28097 project prevented their meaningful stratification into two categories.

## 5. Conclusions

Our data indicate that intraspecific homeostasis of *E. coli* depends on positive correlations among all phylogroups, which are typically maintained at approximately the same level across microbiomes. Intraspecific balance is highly sensitive to host physiological state. External perturbations, compositional shift in biota, or chronic diseases induced adaptive diversification in the abundance of individual phylogroups, which form intraspecific connections but never establish antagonistic relations. Correlation analysis revealed phylogroup-specific differences in interspecific connectivity networks with dominant taxa across all model datasets, consistent with the proposal that intraspecific homeostasis depends on epistatic relationships between *E. coli* phylogroups and evolutionary tuned regulatory networks formed with other genera. The amazing sensitivity of intraspecific homeostasis to both chronic abnormalities and artificial interventions allowed us to identify unexpected patterns and formulate a number of questions for further research. In particular, it has become important to understand to what extent does *E. coli* homeostasis depend on enterotype, how widespread is the bimodal correlation of *E. coli* with *Prevotella* and to what extent are phylogroups of other species sensitive to the physiological state of the host? Using profiles of *E. coli* phylogroups, we observed a high predictive power of UMAP clustering in assessing the physiological status of at least healthy donors. The creation of a database containing sets of reference samples of various intestinal pathologies and sets of phylogroup-specific *k*-mers for potentially virulent bacteria will pave the way for the implementation of intraspecific phylotyping in clinical medicine and diagnostics.

## Figures and Tables

**Table 1:** Datasets used for intraspecific taxonomic analysis.

Type of Dataset	Donor Types and Number of Samples		Bioproject
	Types	Number	
Inflammatory bowel diseases	Healthy individuals	65	PRJEB7774 [48]
	Patients with adenoma	49	
	Patients with carcinoma	46	
Antibiotic treatment with or without probiotic recovery	Fifteen healthy donors before antibiotic treatment	92	PRJEB28097 [49,50]
	Twelve donors of the same group during antibiotic treatment	49	
	Seven donors of the same group during self-recovery	42	
	Eight donors of the same goup during recovery with probiotics	43	
Overweight donors before and after diet	Samples from 43 overweight or obese individuals	86	PRJEB33500 [52]
	Samples from the same 43 persons after Mediterranean diet	86	

**Table 2:** Number of genomes in phylogroups and size of sets with unique 18-mers (barcodes).

Phylogroups	Number of Genomes		Number of 18-Mers	
	Set 1	Set 2	Set 1	Set 2
A	17	21	415,335	354,997
B1	25	25	710,784	524,927
B2	23	29	1,014,716	783,899
C	14	17	242,272	170,224
D	11	15	673,338	524,936
E	13	19	680,604	802,163
F	11	15	445,835	313,383
G	10	13	254,624	171,176

## Data Availability

Sets with unique 18-mers of eight *E. coli* phylogroups are available in the GitHub repository located at https://github.com/marsfro/ecoli_counter (accessed on 6 June 2025). A Python script kmers_ecoli_counter.py developed for counting reads containing phylogroup-specific *k*-mers in WGS metagenomes is available at https://github.com/marsfro/ecoli_counter (accessed on 6 June 2025).

## Supplementary Materials

The following supporting information can be downloaded at https://www.mdpi.com/article/10.3390/microorganisms13071584/s1, Figure S1: Phylogenetic tree of 154 *E. coli* strains constructed with the Set 2 barcodes using the neighbor-joining method. Table S1: *E. coli* strains whose genomes were used for intraspecific phylotyping (Set 2). Table S2: Statistics and normalized percentage of reads with *E. coli* phylogroup-specific 18-mers in human gut microbiomes; Table S3: Intraspecific and interspecific relationships of *E. coli* phylogroups in the human gut microbiomes.
