# Supplementary material for: Phylogroup Homeostasis of Escherichia coli in the Human Gut Reflects the Physiological State of the Host
Source: Microorganisms. 2025 Jul 4;13(7):1584. doi: 10.3390/microorganisms13071584 (PMC12299893; doi:10.3390/microorganisms13071584)
Supplement: Supplementary file 1 [file microorganisms-13-01584-s001.zip › Supplementary Figure S1.pdf]

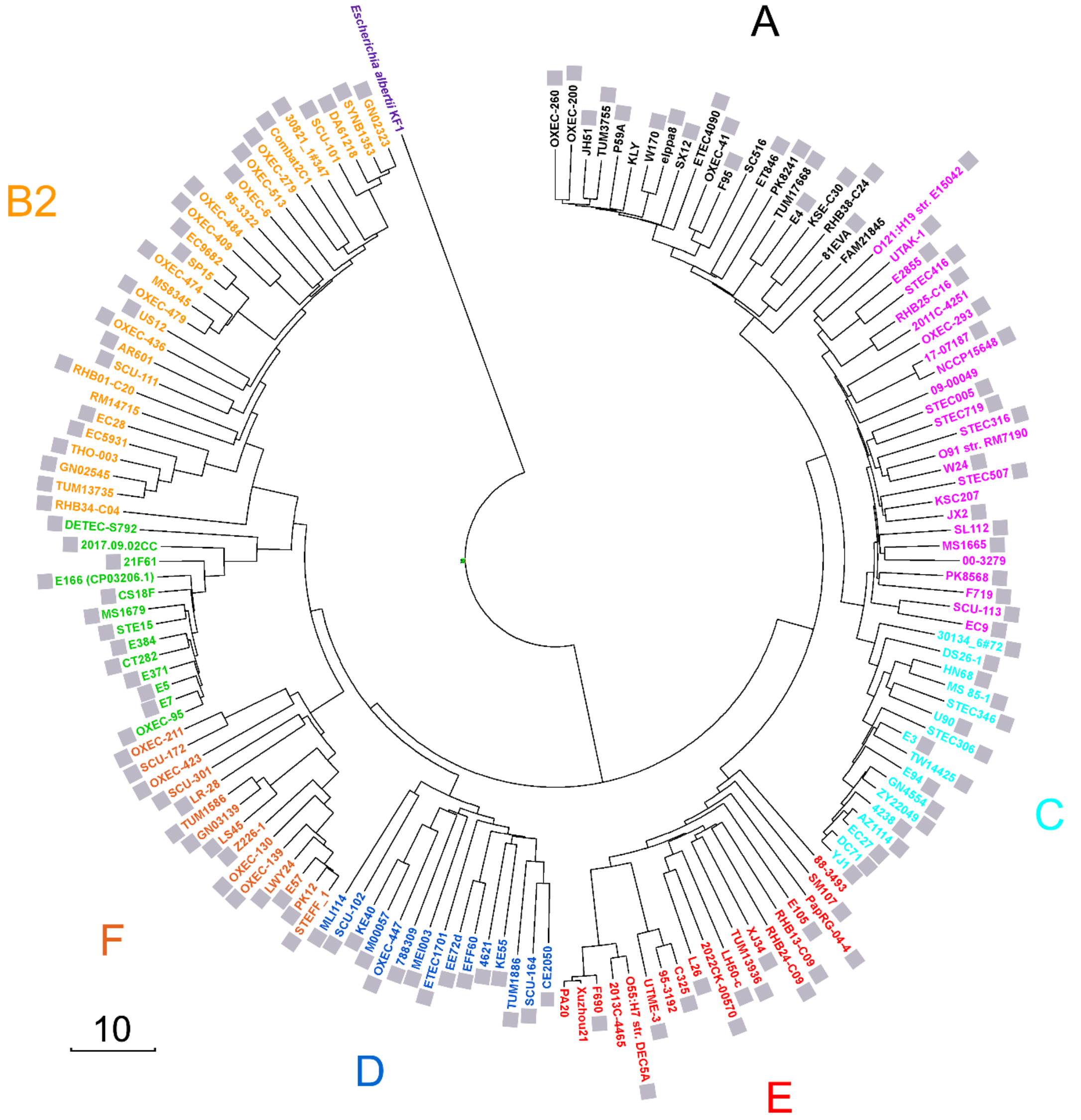

Figure 1. Phylogenetic tree of 154 *E. coli* strains (Set 2) constructed using the neighbor-joining method [53] in MEGA X [54] based on a pairwise distance matrix derived from sets of represetative 18-mers. The scale bar shows the Sørensen distance (percentage). The eight *E. coli* phylogroups are color-coded. A set of representative 18-mers of *Escherichia albertii* KF1 was used as an outgroup. Strains with discordant phylotyping (B1 vs. C) relative to [11] are highlighted with colored circles, while strains not analyzed in [11] are denoted as gray squares.
