# Supplementary material for: Phylogroup Homeostasis of Escherichia coli in the Human Gut Reflects the Physiological State of the Host
Source: Microorganisms. 2025 Jul 4;13(7):1584. doi: 10.3390/microorganisms13071584 (PMC12299893; doi:10.3390/microorganisms13071584)
Supplement: Supplementary file 1 [file microorganisms-13-01584-s001.zip › Supplementary Table S1.pdf]

Supplementary Table S1. *Escherichia coli* strains whose genomes were used for intraspecific phylotyping (Set 2).

Strain	Accession number	Phylogroup	Strain	Accession number	Phylogroup
KLY	CP008801.1	A	YJ1	AP023224.1	C
FAM21845	CP017220.1	A	DS26-1	CP035879.1	C
SC516	CP025048.1	A	EC27	CP060873.1	C
P59A	CP044298.1	A	U90	CP068035.1	C
RHB38-C24	CP057075.1	A	STEC306	CP091016.1	C
PK8241	CP080139.1	A	STEC346	CP091044.1	C
elppa8	CP083492.1	A	DC71	CP101615.1	C
JH51	CP095454.1	A	GN4554	CP102694.1	C
ET846	CP100895.1	A	ZY22049	CP103329.1	C
E4	CP104516.1	A	4238	CP103533.1	C
ETEC4090	CP122620.1	A	E3	CP104536.1	C
KSE-C30	CP124988.1	A	E94	CP119740.1	C
TUM17668	CP134353.1	A	TW14425	CP127265.1	C
TUM3755	CP135680.1	A	AZ1114	CP135204.1	C
81EVA	CP138397.1	A	HN68	CP141844.1	C
SX12	CP142783.1	A	MS 85-1	CP146650.1	C
W170	CP163029.1	A	30134_6#72	OZ038716.1	C
OXEC-41	CP163675.1	A	F690	AP026080.1	E
OXEC-200	CP164286.1	A	Xuzhou21	CP001925.1	E
OXEC-260	CP165072.1	A	2013C-4465	CP015241.1	E
F95	OY754453.1	A	PA20	CP017669.1	E
E2855	AP018796.1	B1	95-3192	CP027362.1	E
O121:H19 str. E15042	AP024478.1	B1	88-3493	CP027457.1	E
NCCP15648	CP009050.1	B1	O55:H7 str. DEC5A	CP038394.1	E
09-00049	CP015228.1	B1	PapRG-04-4	CP049201.1	E
O91 str. RM7190	CP015244.1	B1	RHB24-C09	CP057500.1	E
KSC207	CP019558.1	B1	RHB13-C09	CP057838.1	E
00-3279	CP024293.1	B1	E105	CP071375.1	E
2011C-4251	CP027388.1	B1	XJ34	CP098231.1	E
STEC719	CP041411.1	B1	LH50-c	CP100500.1	E
STEC316	CP041431.1	B1	2022CK-00570	CP114369.1	E
STEC005	CP041437.1	B1	C325	CP124819.1	E
SL112	CP043486.1	B1	SM107	CP130667.1	E
SCU-113	CP051765.1	B1	TUM13936	CP134358.1	E
RHB25-C16	CP057473.1	B1	L26	CP146250.1	E
EC9	CP060950.1	B1	UTME-3	CP168984.1	E
STEC507	CP061238.1	B1	TUM1886	AP026454.1	D
STEC416	CP061241.1	B1	SCU-102	CP051753.1	D
PK8568	CP080126.1	B1	SCU-164	CP054343.1	D
17-07187	CP096976.1	B1	M00057	CP061339.1	D
MS1665	CP097721.1	B1	EE72d	CP065136.1	D
W24	CP141591.1	B1	MEI003	CP071263.1	D
UTAK-1	CP157165.1	B1	CE2050	CP073621.1	D
OXEC-293	CP165507.1	B1	EFF60	CP086678.1	D
JX2	CP173160.1	B1	788309	CP091391.1	D
F719	OY754392.1	B1	4621	CP103479.1	D

MLI114	CP117013.1	D	THO-003	AP022525.1	B2
ETEC1701	CP122938.1	D	SP15	AP024131.1	B2
KE55	CP141061.1	D	TUM13735	AP026514.1	B2
KE40	CP141100.1	D	Combat2C1	CP019243.1	B2
OXEC-447	CP165429.1	D	MS8345	CP025401.1	B2
LR-28	AP027512.1	F	RM14715	CP027104.1	B2
STEFF_1	CP048609.1	F	95-3322	CP027461.1	B2
SCU-301	CP051751.1	F	GN02545	CP041544.1	B2
SCU-172	CP054353.1	F	US12	CP048863.1	B2
LWY24	CP054556.1	F	SCU-101	CP048920.1	B2
PK12	CP074031.1	F	EC28	CP049101.1	B2
LS45	CP095448.1	F	SCU-111	CP051727.1	B2
E57	CP121153.1	F	RHB01-C20	CP055981.1	B2
TUM1586	CP135712.1	F	RHB34-C04	CP057178.1	B2
GN03139	CP147532.1	F	DA61218	CP061206.1	B2
Z226-1	CP159652.1	F	EC9682	CP095271.1	B2
OXEC-139	CP163868.1	F	GN02323	CP095514.1	B2
OXEC-130	CP163898.1	F	EC5931	CP104592.1	B2
OXEC-211	CP164250.1	F	SYNB1353	CP126317.1	B2
OXEC-423	CP164539.1	F	AR601	CP159994.1	B2
2017.09.02CC	AP025214.2	G	OXEC-6	CP163817.1	B2
E166	CP032066.1	G	OXEC-436	CP164490.1	B2
CS18F	CP074576.1	G	OXEC-279	CP164993.1	B2
CT282	CP095798.1	G	OXEC-484	CP165288.1	B2
E371	CP095806.1	G	OXEC-479	CP165308.1	B2
E384	CP095811.1	G	OXEC-474	CP165335.1	B2
STE15	CP095823.1	G	OXEC-513	CP165460.1	B2
MS1679	CP097719.1	G	OXEC-409	CP165468.1	B2
E5	CP104521.1	G	30821_1#347	OZ041026.1	B2
E7	CP104523.1	G			
DETEC-S792	CP116067.1	G			
21F61	CP147038.1	G			
OXEC-95	CP164035.1	G			
